# Isoginkgetin-loaded reactive oxygen species scavenging nanoparticles ameliorate intervertebral disc degeneration via enhancing autophagy in nucleus pulposus cells

**DOI:** 10.1186/s12951-023-01856-9

**Published:** 2023-03-21

**Authors:** Hao Yu, Yun Teng, Jun Ge, Ming Yang, Haifeng Xie, Tianyi Wu, Qi Yan, Mengting Jia, Qing Zhu, Yanping Shen, Lianxue Zhang, Jun Zou

**Affiliations:** 1 Department of Orthopaedic Surgery, The First Affiliated Hospital of Soochow University, 188 Shizi St., Suzhou, 215006 Jiangsu China; 2 Department of Nephrology, The First Affiliated Hospital of Soochow University, 188 Shizi St., Suzhou, 215006 Jiangsu China; 3 College of Life Sciences, Zhejiang University, 866 Yuhangtang Rd., Hangzhou, 310027 Zhejiang China

**Keywords:** Diselenide-containing nanoparticles, Reactive oxygen species (ROS)-responsive drug release, Isoginkgetin, Autophagy, Intervertebral disc degeneration

## Abstract

Excessive reactive oxygen species (ROS) in nucleus pulposus cells (NPCs) promote extracellular matrix (ECM) degradation and cellular inflammatory responses by activating a variety of cellular pathways, ultimately inducing cell apoptosis and leading to the development of low back pain. Here, we designed and fabricated an isoginkgetin-loaded ROS-responsive delivery system (IGK@SeNP) based on diselenide block copolymers. Successfully encapsulated IGK was released intelligently and rapidly in a microenvironment with high ROS levels in degenerative disc. Controlled-release IGK not only efficiently scavenged ROS from the intervertebral disc together with diselenide block copolymers but also effectively enhanced autophagy in NPCs to inhibit ECM degradation and cell apoptosis, and showed significant therapeutic effects in the rat intervertebral disc degeneration (IDD) model. Overall, the synergistic effects of IGK@SeNP in ROS scavenging and autophagy enhancement endowed it with an attractive therapeutic strategy for IDD treatment.

**Supplementary Information:**

The online version contains supplementary material available at 10.1186/s12951-023-01856-9.

## Introduction

Low back pain caused by intervertebral disc degeneration (IDD) has become an important public health concern [1, 2]. In degenerated discs, excessive ROS promote organelle damage, extracellular matrix (ECM) degradation, and cellular inflammatory responses by activating various pathways, ultimately inducing functional cell death in the disc, causing spinal motor unit instability and radicular pain [3–7]. As there are no blood vessels in the intervertebral disc, the concentration of drugs in the intervertebral disc is low, and the efficacy is poor when administered systemically [8, 9]. Currently, the commonly used conservative treatment for IDD is pharmacological analgesia and dehydration [10]. However, these methods can only temporarily relieve pain and eliminate nerve swelling, but cannot remove excessive ROS to delay the progress of IDD, let alone achieve the purpose of curing IDD.

The intervertebral disc consists of a gelatinous inner nucleus pulposus and tough outer annulus fibrosus. The nucleus pulposus is the site of initial degeneration caused by oxidative stress [11]. Early recognition and removal of excessive ROS is a viable therapeutic strategy [12, 13]. For instance, Yang et al. [14] designed an injectable composite hydrogel, which sustained the release of Prussian blue nanoparticles to clear excessive ROS and showed good therapeutic effects in a rat IDD model. Although this strategy can effectively remove excessive ROS, nucleus pulposus cells (NPCs) cannot completely repair oxidative damaged biomolecules and organelles, which seriously affects the metabolic balance and cell viability, and may eventually lead to apoptosis [15, 16]. Therefore, it is necessary to develop a new therapeutic strategy that can not only remove too much ROS in NPCs but also promote the rapid repair of oxidative damage.

When NPCs are subjected to oxidative stress, cells can repair themselves by regulating the level of autophagy to breakdown damaged cellular components [17]. However, when ROS are cleared, cellular autophagy is downregulated, and cells cannot effectively clear damaged organelles, which may cause the cells to undergo apoptosis. Several studies have shown that upregulation of autophagy in NPCs can promote the repair of oxidative damage and reduce apoptosis [18–20]. Isoginkgetin (IGK), a natural flavonoid extracted from ginkgo biloba leaves, can enhance autophagy and scavenge ROS [21, 22]. We believe that it may be a potential drug for the treatment of IDD.

**Scheme 1:**
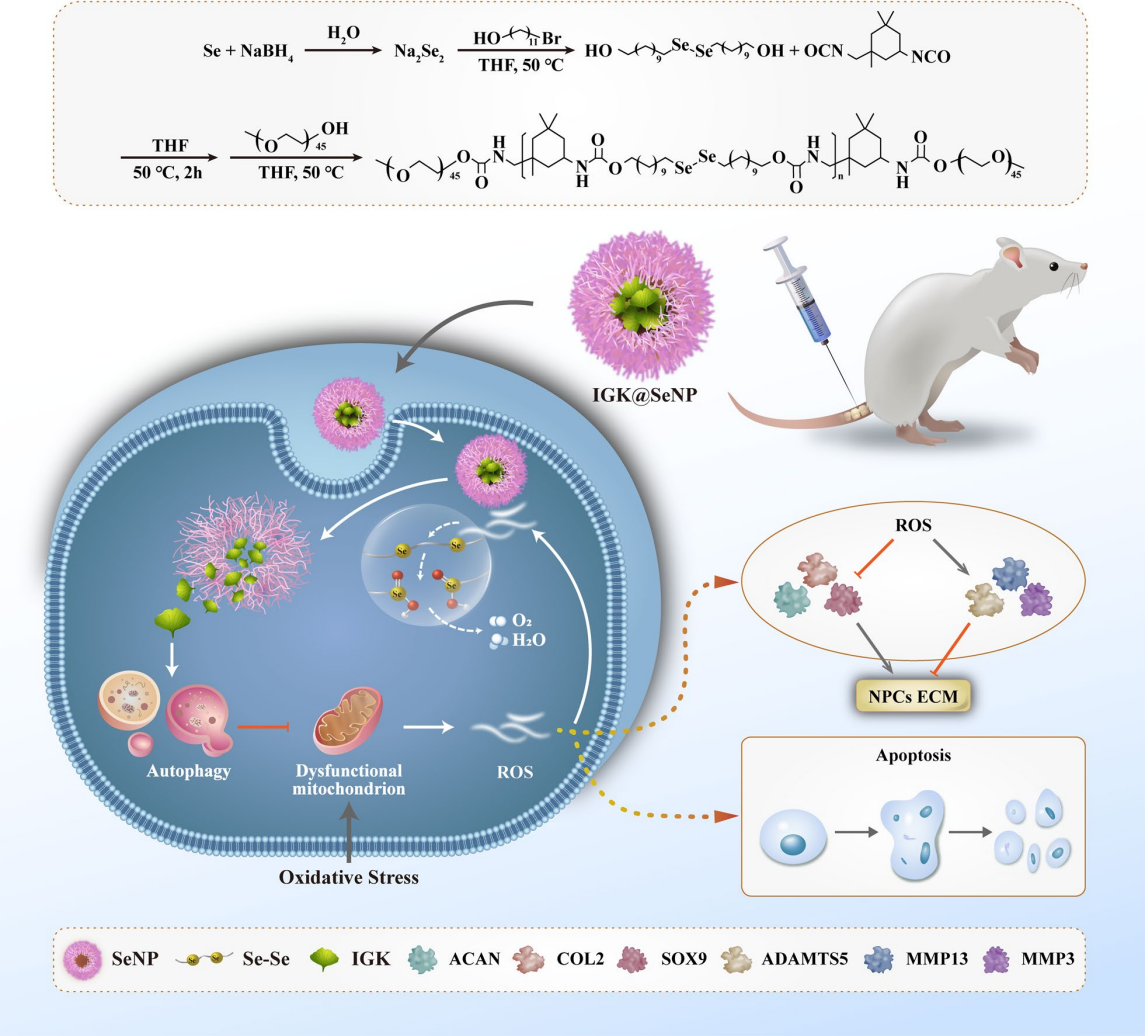
Diagram of IGK@SeNP for the treatment of IDD. A ginkgo biloba extract, IGK, is loaded in Se-Se bond-containing nanoparticles (SeNP). IGK@SeNP is easily taken up by NPCs, and then SeNP scavenges ROS and releases IGK to enhance autophagy, which synergistically protects the ECM of NPCs and reduces cell apoptosis under oxidative stress

In this study, we designed and prepared an ROS-responsive IGK-loaded nanodelivery system (IGK@SeNP) that contained Se-Se bonds and showed good biocompatibility. The encapsulated IGK was intelligently released under the stimulation of high levels of ROS in the degenerative intervertebral disc. Under the combined treatment of IGK and Se-Se bonds, intracellular ROS was effectively cleared. Moreover, controlled-release IGK increased the autophagy level in NPCs, which balanced the anabolism and catabolism of ECM, inhibited apoptosis, and showed a significant therapeutic effect on IDD in vivo (Scheme 1). The strategy of removing intracellular ROS and enhancing autophagy to repair oxidative damage provides a safe and effective novel treatment for IDD.

## Results

### Construction and characterization of IGK@SeNP

**Fig. 1:**
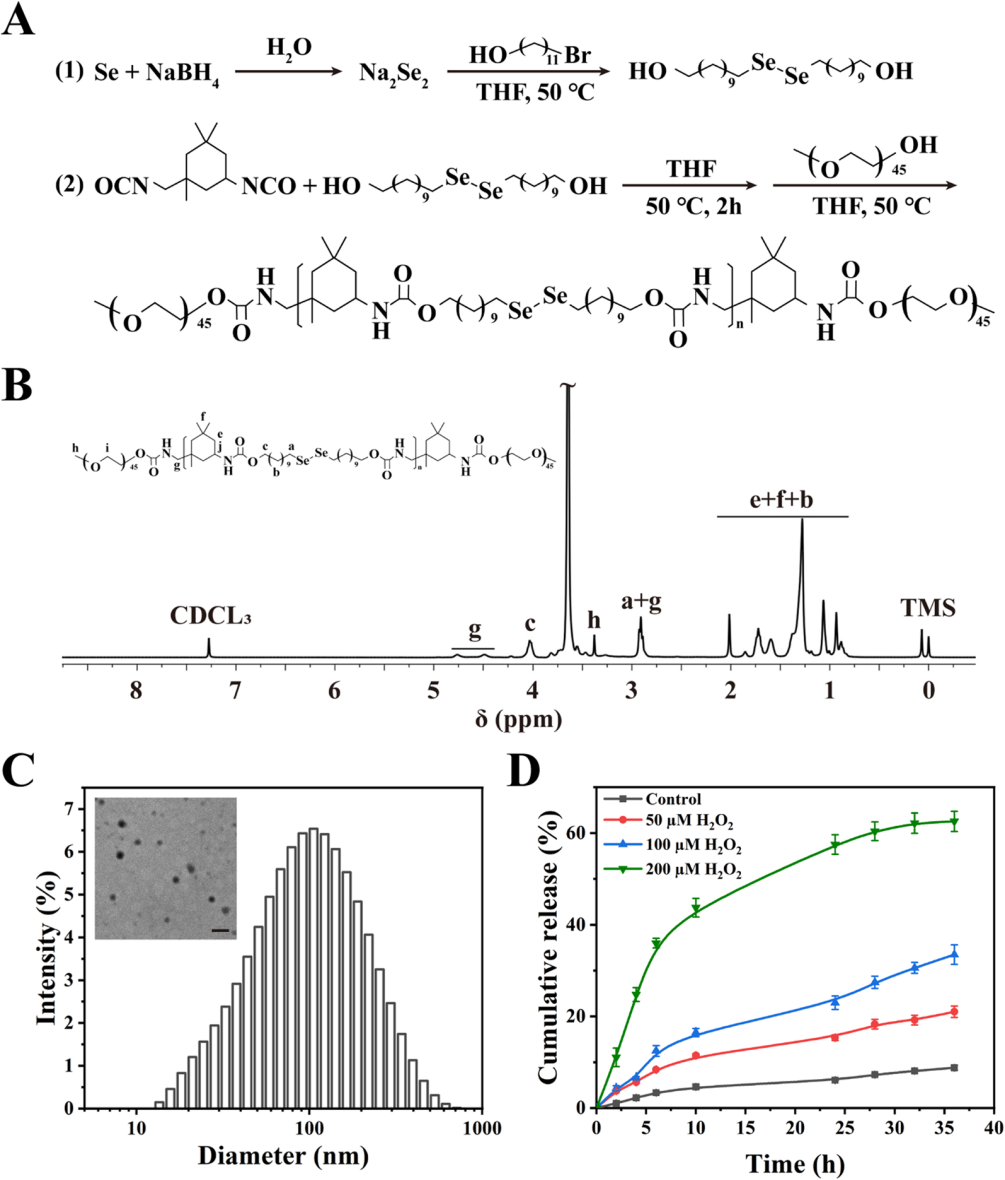
Construction and characterization of SeNP. **A** Synthetic route of diselenide-containing polymer. **B** ^1^H NMR characterization of diselenide-containing polymer. **C** TEM image and size distribution of SeNP. Scale bar = 200 nm. **D** Cumulative drug release curves of Cy5@SeNP at different concentrations of H_2_O_2_. n = 3, mean ± SD

The diselenide-containing polymer (Se polymer) was synthesized according to our previous study (Fig. 1A). Briefly, di(1-hydroxylundecyl) diselenide was first synthesized, polymerized with isophorone diisocyanate (IPDI), and finally reacted with methoxypolyethylene glycol (mPEG). The ^1^H NMR spectrum demonstrated the successful synthesis of di(1-hydroxylundecyl) diselenide (Additional file 1: Fig. S1) and Se-polymer (Fig. 1B). GPC analysis determined the molecular weight of the Se-polymer to be 11950 g/mol (Additional file 1: Fig. S2).

Owing to its amphiphilicity, the Se-polymer can self-assemble into nanoparticles (SeNP) in aqueous solution. The amphiphilic Se-polymer and hydrophobic IGK self-assembled into micelle nanoparticles in aqueous solution (hereafter referred to as IGK@SeNP). Transmission electron microscopy (TEM) revealed that the IGK@SeNP possessed a spherical morphology with an average diameter of approximately 100 nm (Fig. 1C). Dynamic light scattering (DLS) indicated that the average hydrodynamic diameter was approximately 108 nm, with a polydispersity index of 0.25.

### In vitro* drug release*

The in vitro ROS-responsive drug release behavior of the nanoparticles was investigated. Insoluble Cy5 was loaded as the model cargo given its easy detectability (named Cy5@SeNP hereafter). Different concentrations of hydrogen peroxide (H_2_O_2_) divided this experiment into four groups: (a) PBS control group, (b) 50 μM H_2_O_2_ co-treatment group, (c) 100 μM H_2_O_2_ co-treatment group, and (d) 200 μM H_2_O_2_ co-treatment group.

As shown in Fig. 1D, in the control group, Cy5@SeNP achieved a drug release rate of only ~ 9% at 36 h, which indicated that Cy5@SeNP had favorable stability under physiological conditions. In contrast, in the three H_2_O_2_ treatment groups, the release rate significantly increased with increasing concentrations of H_2_O_2_. For example, under the stimulation of 200 μM H_2_O_2_, the drug was released rapidly, with a cumulative release percentage of  ~ 63% at 36 h. The above results suggest that SeNP exhibited an excellent property of controlled release in the presence of ROS. Therefore, we expect SeNP to release drugs to treat IDD under oxidative conditions.

### *Biocompatibility and ROS-scavenging properties *in vitro

**Fig. 2:**
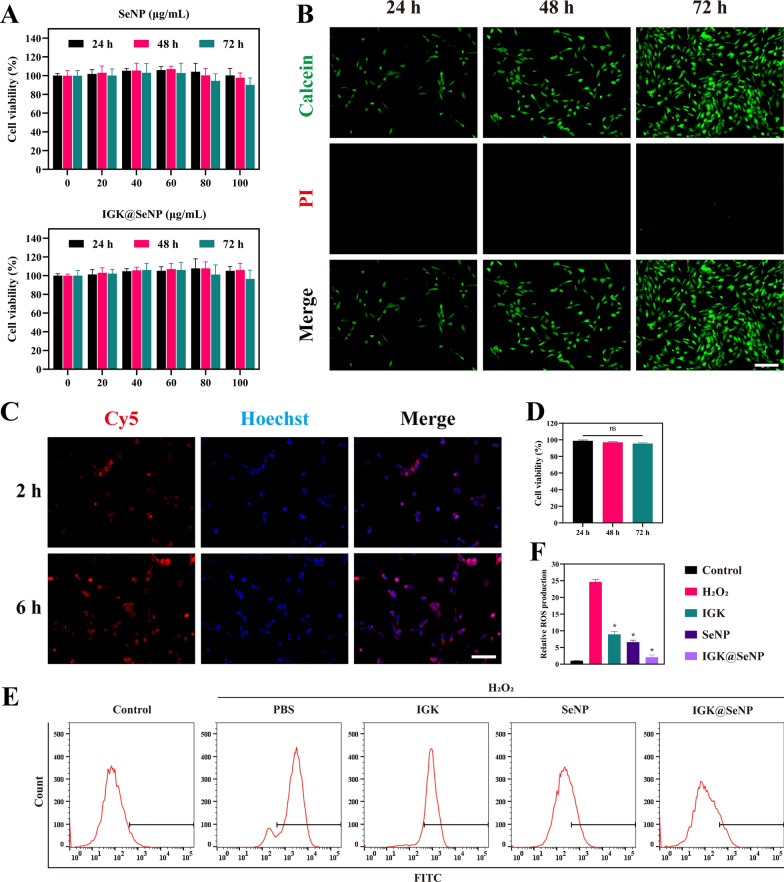
Biocompatibility and ROS scavenging properties in vitro. **A** CCK-8 assay was used to evaluate the cell cytotoxicity of SeNP and IGK@SeNP against NPCs after incubation for 24 h, 48 h, and 72 h. n = 6, mean ± SD. **B, D** Live/dead staining and cell viability of NPCs co-cultured with IGK@SeNP (60 μg/ml) for 24 h, 48 h, and 72 h. Scale bar = 200 μm. n = 3, mean ± SD. ns, not significant. **C** Fluorograph of the cellular uptake of Cy5@SeNP by NPCs. Scale bar = 100 μm. **E**, **F** Flow cytometry analysis and relative geometric mean fluorescence intensity of intracellular ROS levels in NPCs treated with H_2_O_2_ (200 μM) and different treatments. n = 3, mean ± SD. *p < 0.05 compared to the H_2_O_2_ group

To determine the biocompatibility of the nanoparticles, we first treated NPCs with various concentrations of SeNP. The CCK-8 assay results showed that SeNP at 0–60 μg/ml had no significant toxicity on NPCs within 72 h (Fig. 2A). Therefore, we chose the concentration of SeNP to be 60 μg/ml in the subsequent study. IGK@SeNP at the above concentrations were co-incubated with NPCs for 72 h to detect their biocompatibility. Live/dead staining indicated that NPCs maintained good growth ability after co-culture with IGK@SeNP for 72 h, and the percentage of viable cells was not statistically different from that in the 24 h group (Fig. 2B, D). These results showed that the IGK@SeNP had excellent compatibility with NPCs.

Cy5 was used as a drug model to assess cellular internalization of the nanocarriers. As shown in Fig. 2C, when NPCs were co-cultured with Cy5@SeNP (red) for 2 h, red fluorescence was observed in cells, indicating that Cy5@SeNP was internalized into NPCs. After 6 h of culture, the red fluorescence intensity was significantly enhanced, indicating that more Cy5@SeNP were internalized by the NPCs. These results indicate that SeNP can be effectively internalized by NPCs.

To verify the ROS scavenging ability of IGK, SeNP, and IGK@SeNP, we established a cell model of oxidative stress in NPCs by co-incubating the NPCs with 200 μM H_2_O_2_ for 24 h and calculated the intracellular ROS content using geometric mean fluorescence intensity (gMFI). We found that intracellular ROS levels in the H_2_O_2_ group (gMFI = 1891 ± 53) increased 21-fold compared with the control group (gMFI = 77 ± 3). Subsequently, different NPC treatments were administered. As shown in Fig. 2E, F, IGK decreased intracellular ROS levels (gMFI = 683 ± 66), but its effect was weaker than that of SeNP (gMFI = 506 ± 44). In contrast, IGK@SeNP exhibited the strongest antioxidant capacity (158 ± 52). These results indicate that IGK@SeNP has an excellent synergistic effect in eliminating excessive ROS in NPC.

### Anti‑ECM degradation effects of IGK@SeNP on NPCs

**Fig. 3:**
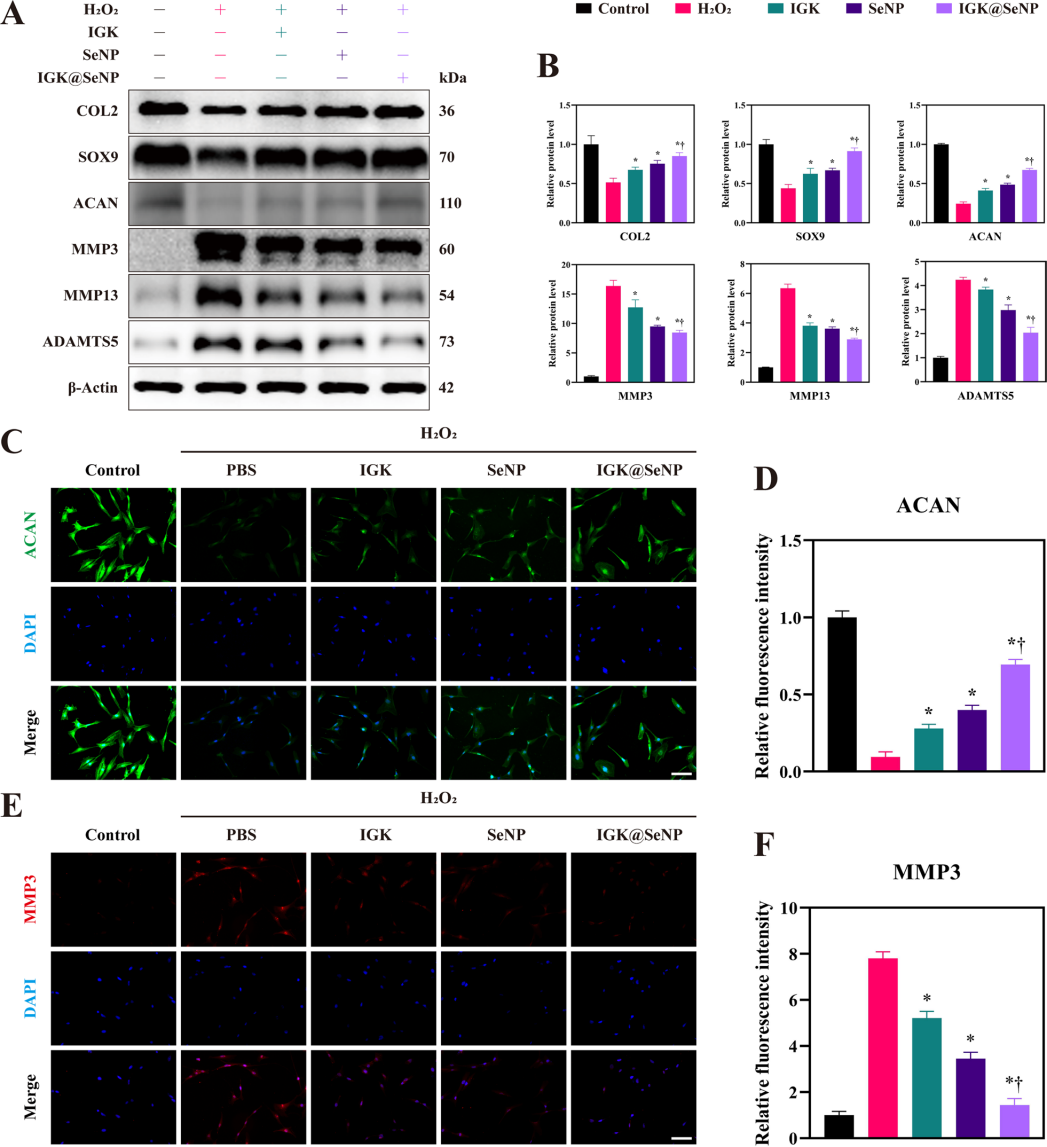
IGK@SeNP attenuates H_2_O_2_-induced ECM degradation. **A** Western blotting analysis of the expression of ECM anabolic proteins (COL2, SOX9, and ACAN) and ECM catabolic enzymes (MMP3, MMP13, and ADAMTS5) in 200 μM H_2_O_2_-induced NPCs treated with IGK (20 μM), SeNP (60 μg/ml), and IGK@SeNP (60 μg/ml). **B** Densitometric analysis of ECM metabolism-related protein levels. **C**–**F** IF staining of ACAN and MMP3 in NPCs. Scale bar = 100 μm. n = 3, mean ± SD. *p < 0.05 compared to the H_2_O_2_ group, †p < 0.05 compared to the IGK group

Western blotting was performed to evaluate the protective effect of IGK@SeNP on ECM (Fig. 3A, B). H_2_O_2_ treatment decreased the expression of the ECM anabolic proteins COL2, SOX9, and ACAN and increased the expression of the ECM catabolic enzymes MMP3, MMP13, and ADAMTS5. These changes indicate that oxidative stress tilts the metabolic balance of the ECM toward catabolism. Supplementation with IGK, SeNP, or IGK@SeNP reversed the disordered ECM metabolism induced by H_2_O_2_. However, the correction of protein expression in the IGK group was slightly weaker than that in the SeNP group, which may be due to the poor water solubility of IGK and its difficulty in entering cells to exert biological effects. Notably, in the IGK@SeNP group, the expression of ECM metabolism-related proteins was significantly restored, with a better effect than that in the IGK and SeNP groups. Furthermore, immunofluorescence (IF) staining was conducted for the representative component (ACAN) and stromelysin (MMP3) in the nucleus pulposus (Fig. 3C–F). Consistent with previous results, the IGK@SeNP group most significantly increased the fluorescence intensity of ACAN and decreased the fluorescence intensity of MMP3. Collectively, the above data showed that H_2_O_2_-induced oxidative stress can disrupt the metabolic balance of ECM, while the combination of SeNP and IGK can better regulate the anabolism and catabolism of ECM than individual treatments.

### Anti‑apoptosis effects of IGK@SeNP on NPCs

**Fig. 4:**
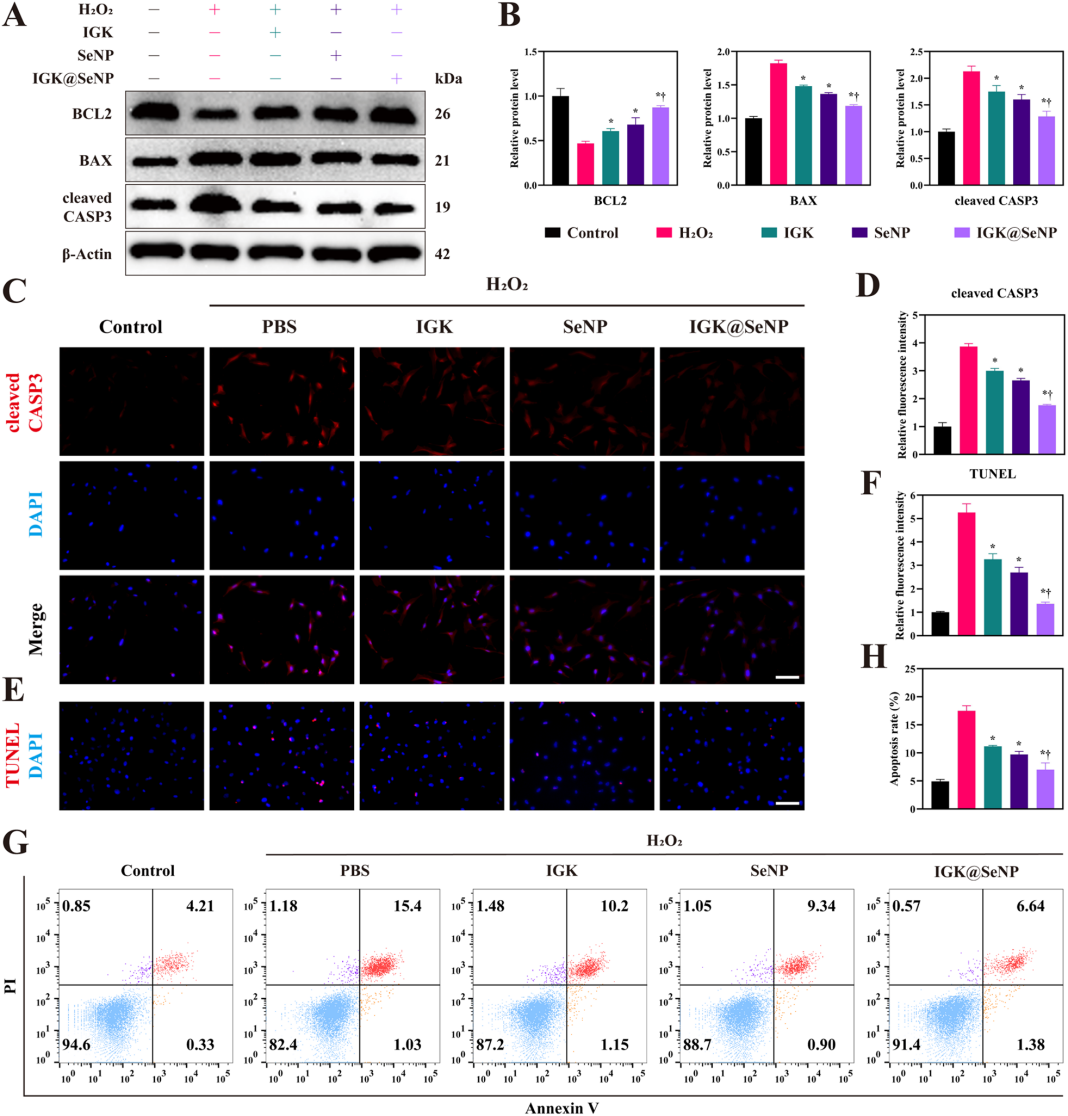
IGK@SeNP inhibits H_2_O_2_-induced NPC apoptosis. **A** Western blotting analysis of the expression of an antiapoptotic protein (BCL2) and pro-apoptosis proteins (BAX and cleaved CASP3). **B** Densitometric analysis of apoptosis-related protein levels. **C**, **D** IF staining of cleaved CASP3 in NPCs. Scale bar = 100 μm. **E**, **F** TUNEL staining of NPCs after different treatments. Scale bar = 100 μm. **G**, **H** Flow cytometry analysis with FITC-annexin V/PI assay. *p < 0.05 compared to the H_2_O_2_ group, †p < 0.05 compared to the IGK group

To determine the protective effect of the nanodrug delivery system on NPCs under oxidative stress, western blotting was used to detect changes in the expression of key proteins in the apoptotic pathway (Fig. 4A, B). After treatment with H_2_O_2_ for 24 h, the expression of BCL2 was downregulated, while BAX and cleaved caspase3 (CASP3) were upregulated, indicating that apoptosis was activated by oxidative stress. However, after treatment with IGK, SeNP, and especially IGK@SeNP, the changes in the expression of the above proteins became weaker, indicating that the apoptosis pathway was blocked.

Accumulation of ROS has been demonstrated to increase the activity of CASP3, a critical protein in the apoptosis pathway, leading to amplification of the proteolytic cleavage cascade, ultimately causing cell apoptosis [23]. IF staining showed that the high level of cleaved CASP3 induced by H_2_O_2_ decreased in the IGK, SeNP, and IGK@SeNP groups, in which IGK@SeNP achieved the optimal effect (Fig. 4C, D). TUNEL staining showed increased DNA fragmentation in the H_2_O_2_ group, indicating that the NPCs underwent apoptosis under H_2_O_2_ stimulation (Fig. 4E, F). Fortunately, the number of TUNEL-positive cells decreased in the three experimental groups, and again, the IGK@SeNP group showed the strongest cytoprotection. These results were also confirmed with flow cytometry, showing that the apoptosis rate was 4.93 ± 0.28% in the control groups and 17.49 ± 0.75% in the H_2_O_2_ groups, which was reduced to 11.18 ± 0.14%, 9.71 ± 0.44%, and 7.02 ± 0.98% in the IGK, SeNP, and IGK@SeNP groups, respectively (Fig. 4G, H). The above results indicated that IGK@SeNP could effectively inhibit NPC apoptosis induced by H_2_O_2_.

### Pro-autophagy effects of IGK@SeNP on NPCs

**Fig. 5:**
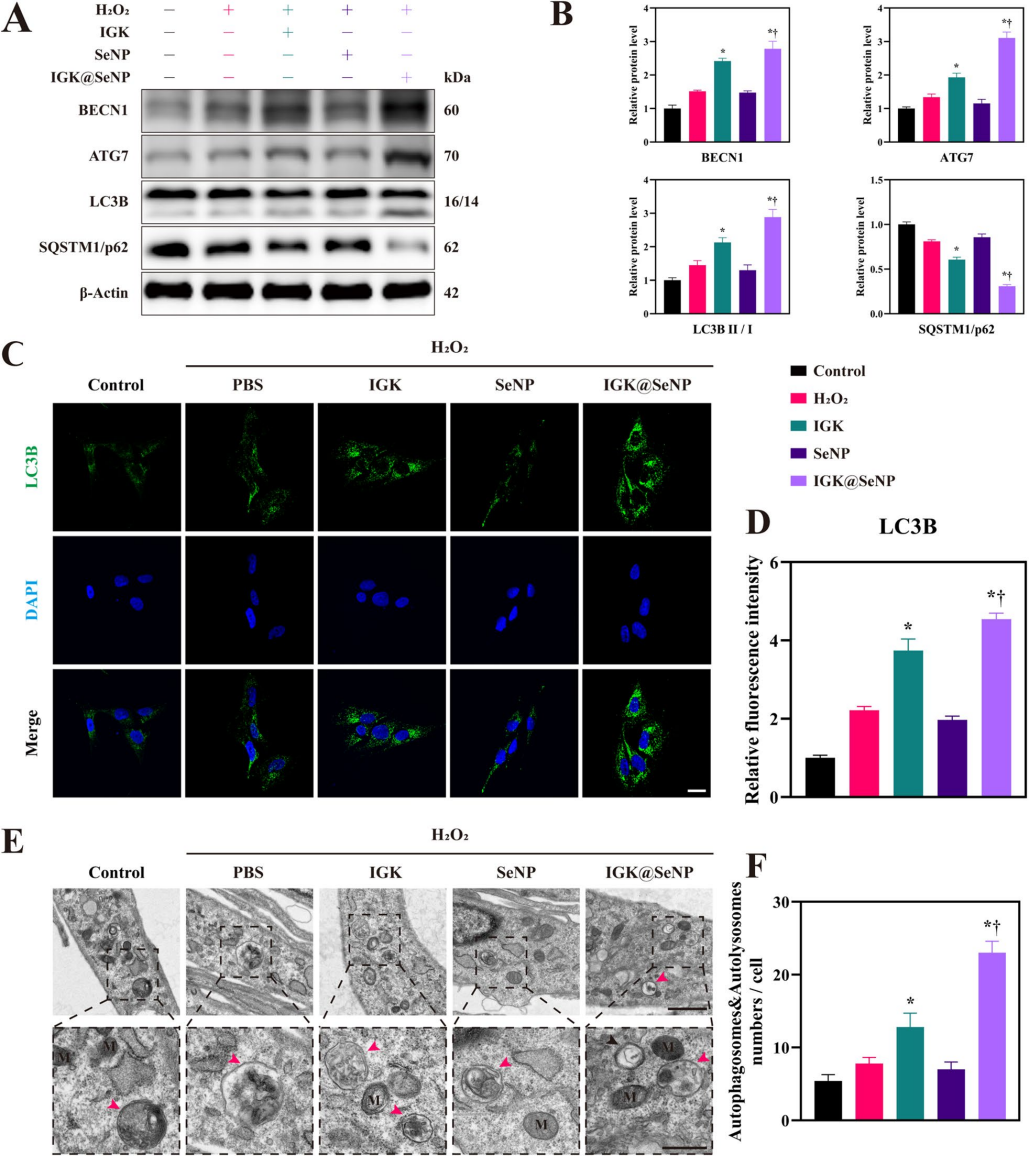
IGK@SeNP promotes autophagy in H_2_O_2_-treated NPCs. **A** Western blotting analysis of the expression of autophagy-related proteins (BECN1, ATG7, LC3B, and SQSTM1/p62). **B** Densitometric analysis of autophagy-related protein levels. **C**, **D** IF staining of LC3B in NPCs incubated with IGK, SeNP, and IGK@SeNP in the presence of H_2_O_2_ stimulation. Scale bar = 20 μm. **E**, **F** TEM analysis of morphology and the number of autophagosomes and autolysosomes in NPCs. Red arrows indicate autolysosomes with single membranes, while black arrows indicate autophagosomes with double membranes. M indicates mitochondria. Scale bar = 1 μm (upper row) and 500 nm (lower row). n = 3, mean ± SD. *p < 0.05 compared to the H_2_O_2_ group, †p < 0.05 compared to the IGK group

Moderate autophagy helps cells survive in harsh environments, whereas excessive autophagy also induces cell death [24–26]. Therefore, we explored the role of autophagy in the process of IGK treatment in IDD. As autophagy is regulated by multiple proteins at different stages, several key indicators are used to reflect changes in autophagic flux. For example, Beclin1 (BECN1) binds to pre-autophagosomes to initiate autophagosome formation. ATG7 participates in the assembly of autophagosomes by promoting the conjugation of ATG12 to ATG5 and the conversion of LC3I to LC3II. Then, LC3II binds to autophagosome membranes and is widely used as a marker for autophagy. Eventually, SQSTM1/p62 delivers autophagic substrates to the autophagosome and is degraded with these substrates in autolysosomes. As shown in Fig. 5A, B, H2O2 activated autophagy, marked by the upregulated expression of BECN1 and ATG7 and the ratio of LC3B II/I, while downregulating the expression of SQSTM1/p62. Treatment with IGK further amplified these changes in the protein levels. IF staining of LC3B also confirmed that there were more punctate structures in the IGK and IGK@SeNP groups, suggesting that the number of autophagosomes combined with LC3B II might be increased (Fig. 5C, D).

Autophagy is a complex process because an increase in LC3II levels may be caused by either enhanced autophagic flux or insufficient autolysosomes [27]. Therefore, we detected autophagy-associated vesicles by TEM. As shown in Fig. 5E, in the H_2_O_2_ group, autolysosomes wrapped the damaged organelles, and mitochondria dissolved into vacuoles, suggesting that oxidative stress damaged mitochondria. However, the dysfunctional mitochondria were not completely cleared, which may have led to their continuous production of ROS and eventually induced apoptosis of NPCs. In addition, a higher number of autophagy-associated vesicles was observed after IGK treatment, especially in the IGK@SeNP group, indicating that the SeNP drug delivery system favored the autophagy-enhancing properties of IGK (Fig. 5F).

We also noticed that the SeNP group showed a slight inhibition of autophagy compared with the H_2_O_2_ group, but interestingly, the IGK-loaded SeNP still showed a more obvious autophagy enhancement effect. These results indicate that the autophagy stimulator IGK was steadily transferred and released from the ROS-responsive SeNP and more effectively enhanced autophagy, thus regulating ECM metabolism and restraining apoptosis of NPCs.

### Effects of autophagy blockers on the therapeutic efficacy of IGK@SeNP

**Fig. 6:**
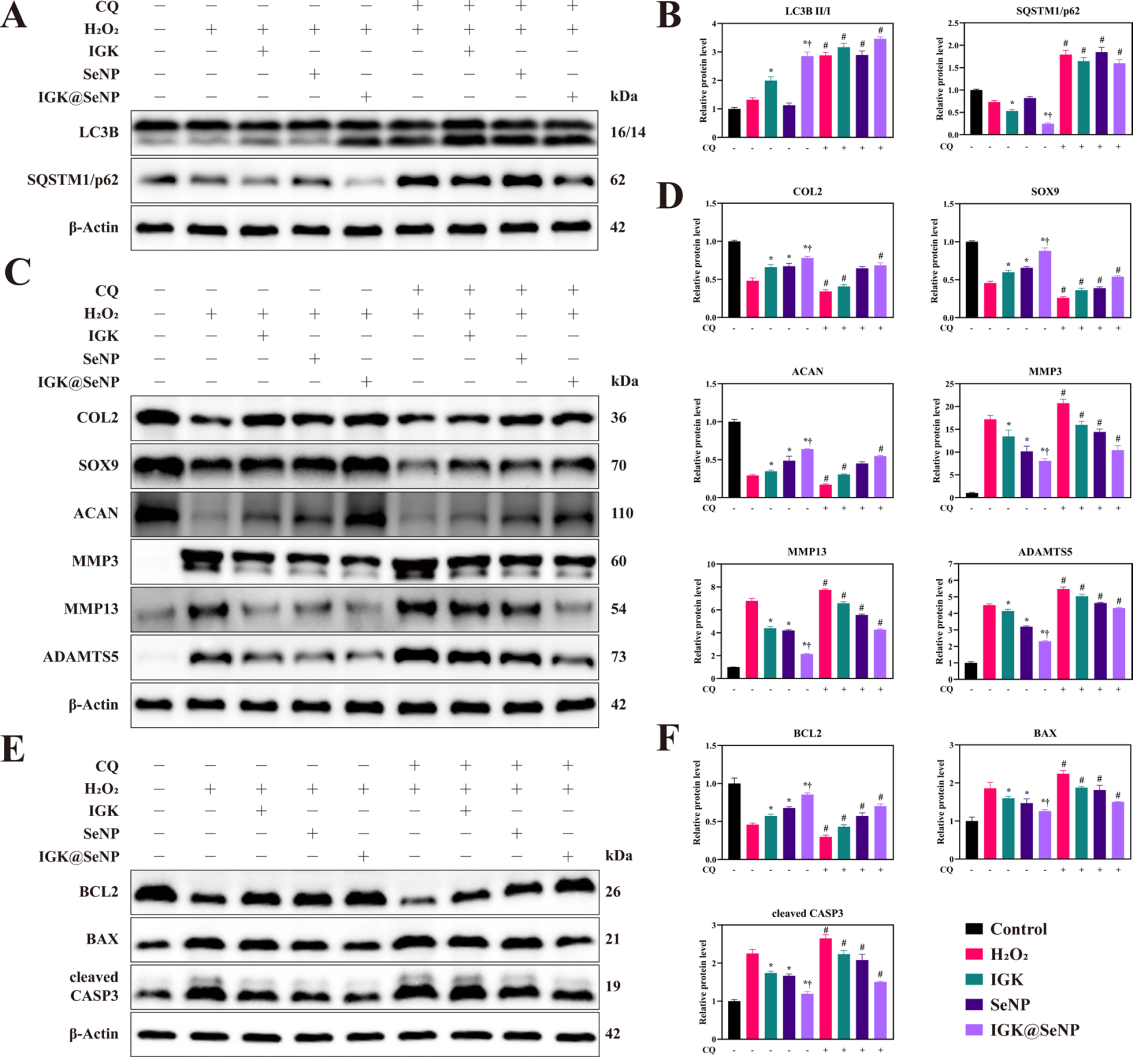
Autophagy blocker reduces the anti-ECM degradation effect and anti-apoptotic effect of IGK@SeNP. **A** Western blotting analysis of the expression of autophagy-related proteins in NPCs induced by H_2_O_2_ with or without CQ (10 μM) for 24 h. **B** Densitometric analysis of autophagy-related protein levels. **C** Western blotting analysis of the expression of ECM metabolism-related proteins in NPCs induced by H_2_O_2_ with or without CQ (10 μM) for 24 h. **D** Densitometric analysis of ECM metabolism-related protein levels. **E** Western blotting analysis of the expression of apoptosis-related proteins in NPCs induced by H_2_O_2_ with or without CQ (10 μM) for 24 h. **F** Densitometric analysis of apoptosis-related protein levels. n = 3, mean ± SD. *p < 0.05 compared to the H_2_O_2_ group, †p < 0.05 compared to the IGK group, #p < 0.05 compared to the same treatment groups without CQ

To confirm that IGK protects NPCs by enhancing autophagy, we treated NPCs with 10 μM chloroquine (CQ), an autophagic blocker that inhibits the fusion of autophagosomes with lysosomes. The protein levels of LC3B and SQSTM1/p62 increased after treatment with CQ, indicating an interrupted autophagic flux (Fig. 6A, B). Moreover, in the presence of CQ, the effect of IGK@SeNP in regulating the expression of ECM metabolism-related proteins (COL2, SOX9, ACAN, MMP3, MMP13, and ADAMTS5) and apoptosis-related proteins (BCL2, BAX, and cleaved CASP3) decreased (Fig. 6C–F). These results further verified that IGK@SeNP protects NPCs by enhancing autophagy under oxidative stress.

### Therapeutic efficacy of IGK@SeNP in the rat IDD model

**Fig. 7:**
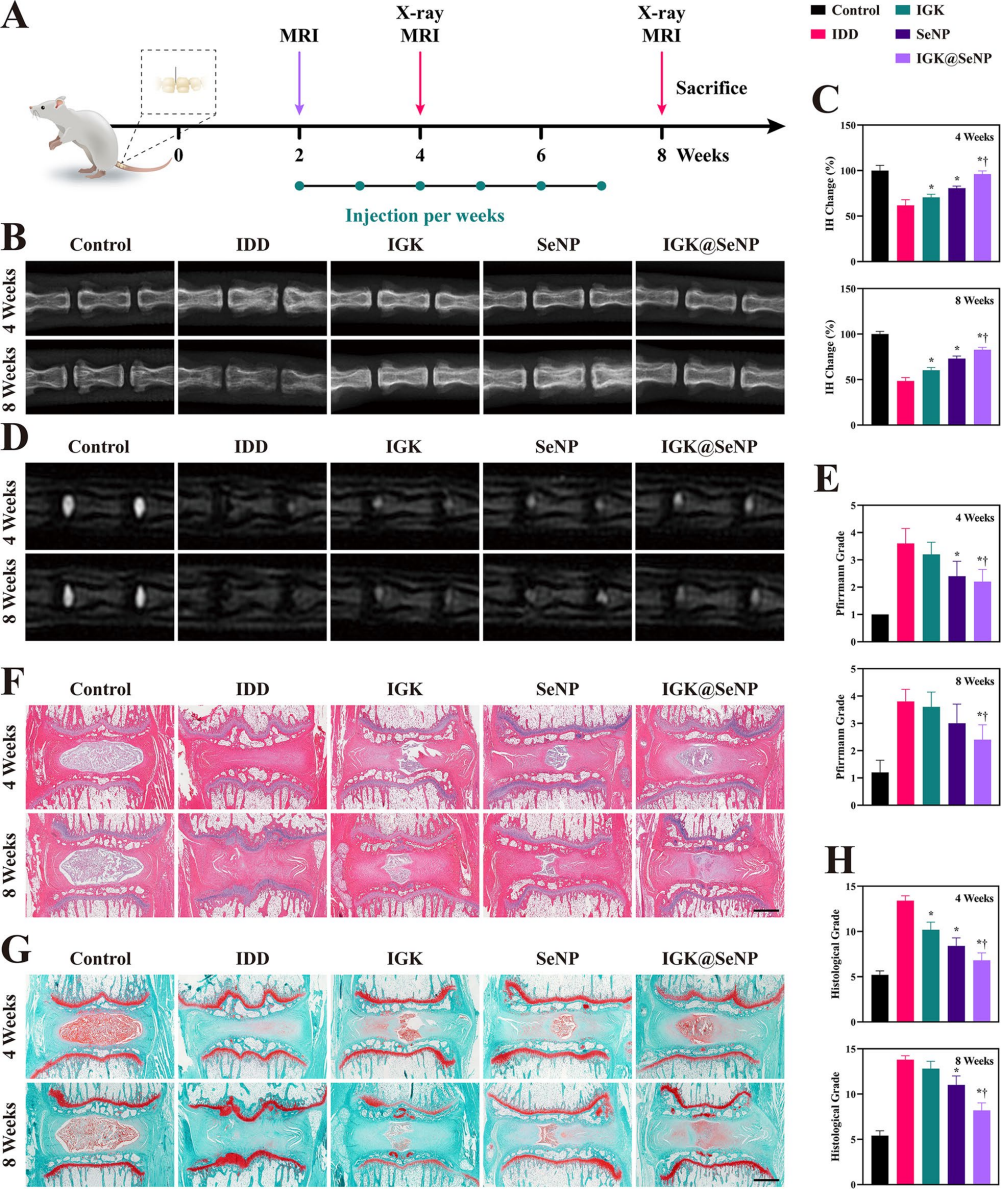
IGK@SeNP protects the nucleus pulposus in needle puncture-induced IDD rats. **A** Treatment schedule of animal experiments. Two weeks after surgery, the IDD model was validated by MRI. PBS, IGK, SeNP, and IGK@SeNP were injected weekly thereafter. Imaging examinations were performed at weeks 4 and 8. **B** X-ray images of rat coccygeal spines at the 4th and 8th-week post-surgery. **C** Intervertebral heights of rat coccygeal spines measured on the X-ray. **D** T2-weighted MRI of coccygeal discs at the 4th and 8th-week post-surgery. **E** Pfirrmann grade scores of coccygeal discs measured on MRI. **F**, **G** HE and SF staining of rat coccygeal discs. Glycosaminoglycans are shown with safranin O. Scale bar = 1 mm. **H** Histological grade scores of coccygeal discs at the 4th and 8th-week post-surgery. n = 5, mean ± SD. *p < 0.05 compared to the IDD group, †p < 0.05 compared to the IGK group

Given the favorable results of in vitro anti-ECM degradation and anti‑apoptosis potential, the efficacy of IGK@SeNP in treating IDD in vivo was further evaluated in a rat model (Fig. 7A). The IDD rat model was established using needle puncture. Degenerative discs were locally injected with PBS, IGK, SeNP, or IGK@SeNP. Imaging examinations, including X-ray and magnetic resonance imaging (MRI), were used to evaluate the degree of disc degeneration 4 and 8 weeks after surgery. As shown in Fig. 7B, C, intervertebral height was significantly preserved in the IGK@SeNP group compared to that in the IDD group. However, the intervertebral height in the IGK group gradually decreased in 8 weeks, indicating that in vivo treatment with IGK alone is not very effective, while combining it with the SeNP drug delivery system enhanced the therapeutic effect. MRI revealed that the T2-weighted signal in the IDD group had declined, indicating a loss of water in these discs (Fig. 7D). After 6 weeks of treatment, the water content was preserved in the IGK@SeNP group, with significantly low signals in the IGK groups. The Pfirrmann grade results further confirmed that IGK@SeNP had superior efficacy for treating IDD in vivo (Fig. 7E).

Histological sections were collected at 4 and 8 weeks after surgery. Hematoxylin–eosin (HE) staining was used to observe the morphology of the nucleus pulposus and annulus fibrosus (Fig. 7F). The nucleus pulposus of the IDD group prominently decreased in volume and was replaced by fibrous tissue at 8 weeks, and the arrangement of the annulus fibrosus was disordered. In addition, a substantial loss of glycosaminoglycans was observed at 4 and 8 weeks in the IDD group by safranin O-fast green (SF) staining (Fig. 7G). Histological changes induced by needle puncture were weakened in the IGK@SeNP group. However, in the IGK and SeNP groups, the loss of the nucleus pulposus was evident with destruction of the disc structure. Based on the histological grade, the IDD group recorded the highest score, whereas the IGK@SeNP group recorded the lowest score, except for the control group (Fig. 7H).

**Fig. 8:**
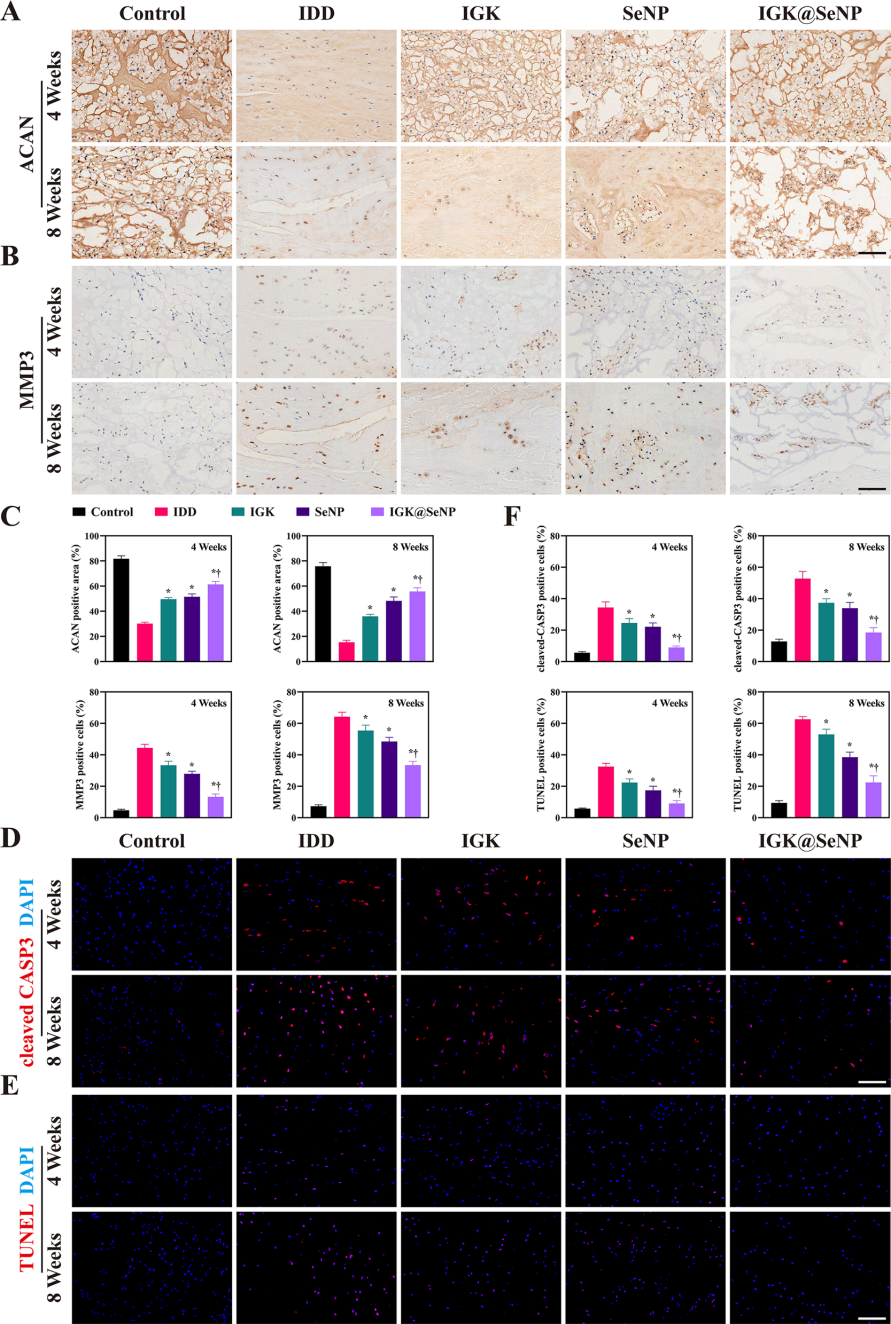
IGK@SeNP attenuates matrix degradation and cell apoptosis in vivo. **A**, **B** IHC staining of ACAN and MMP3 in rat coccygeal discs. **C** Quantitative analysis of ACAN- and MMP3-positive rates in different groups. **D** IF staining of cleaved CASP3 in rat coccygeal discs. **E** TUNEL staining of rat coccygeal discs. **F** Quantitative analysis of cleaved CASP3- and TUNEL-positive cells in different groups. Scale bar = 100 μm. n = 5, mean ± SD. *p < 0.05 compared to the IDD group, †p < 0.05 compared to the IGK group

We performed immunochemical (IHC) staining to analyze the expression levels of ACAN and MMP3, which are representative indices of matrix metabolism in the nucleus pulposus. After needle puncture surgery, the expression of ACAN in all model groups was degraded, while the IGK@SeNP group maintained the highest ACAN expression among the model groups (Fig. 8A). Conversely, the expression of MMP3 was substantially increased in the IDD group and was relatively restrained in the IGK, SeNP, and particularly in the IGK@SeNP group, indicating that the ECM of the nucleus pulposus was rescued (Fig. 8B).

Subsequently, we used TUNEL assay and IF staining of cleaved CASP3 to analyze the apoptosis of NPCs in degenerative discs (Fig. 8D, E). In the IDD group, the apoptosis pathway was upregulated, and the apoptosis rate increased remarkably with time. However, treatment with IGK@SeNP inhibited this pathological progression in vivo. The above results indicate that IGK@SeNP exerts a prominent therapeutic effect on IDD in vivo.

## Discussion

The incidence of IDD increases with age and can lead to spinal instability, radicular pain, and even disability, making it a critical health issue [2, 28]. Many studies have found that ROS accumulation and high levels of oxidative stress are important causes of IDD development because they can cause ECM degradation and NPC death [29, 30]. Therefore, ROS have become a key target for the treatment of IDD. However, traditional therapeutic agents have difficulty accessing the interior of avascular intervertebral discs, so they can only reduce inflammation and temporarily relieve pain outside the intervertebral disc but cannot remove accumulated ROS, which makes them unable to fundamentally prevent the progression of IDD [31, 32]. Although some studies have used novel biomaterials to deliver drugs into the intervertebral disc and effectively reduce ROS levels, oxidatively damaged organelles are not removed simultaneously, which may cause blockage in the repair of cell viability and metabolic function, ultimately affecting the therapeutic effect [14, 33, 34]. Therefore, there is an urgent need to develop new strategies to effectively treat IDD by removing damaged organelles and reducing ROS. In this study, we fabricated an ROS-responsive nanodelivery system based on diselenide block copolymers that can load and intelligently release IGK, a natural autophagy stimulator. Our experiments confirmed that IGK@SeNP could effectively scavenge ROS and delay the progression of IDD in rats by enhancing autophagy to degrade oxidatively damaged mitochondria. Moreover, blocking autophagy attenuated the protective function of IGK@SeNP in regulating ECM metabolism and inhibiting apoptosis, indicating that IGK@SeNP has the dual efficacy of ROS clearance and autophagy enhancement, which makes it advantageous for IDD treatment.

Oxidative stress plays an important role in many physiological processes, such as cell signaling and immune responses. An important mechanism is that ROS can promote the expression of BAX and release cytochrome C by increasing the permeability of the outer mitochondrial membrane, which activates caspase-mediated cascade amplification and promotes apoptosis, resulting in tissue entrapment into a pathological state. Cheng et al. [33] revealed that the combination of antioxidants and thermosensitive hydrogels could decrease oxidative stress induced by H_2_O_2_ and restore the content of GAGs in the early stages of IDD. Bai et al. [35] developed a rapamycin-loaded hydrogel and successfully induced macrophage differentiation into the M2 phenotype, thereby alleviating the inflammatory microenvironment of the intervertebral disc. Zhu et al. [34] used MnO_2_ nanoparticles to control the release of TGF-β3, and this approach suppressed H_2_O_2_-induced oxidative damage by increasing the expression of antioxidative genes. Our study showed that SeNP was able to scavenge ROS owing to the reducibility of Se-Se bonds, which prevented H_2_O_2_-induced ECM degradation and NPC apoptosis. However, this effect was insufficient. We found that although SeNP removed intracellular ROS, the autophagy level decreased, marked by the downregulation of initiation-related proteins (BECN1, ATG7, and LC3B). This deficiency resulted in the inability of NPCs to repair oxidatively damaged organelles, as vacuolization in mitochondria was observed in the SeNP group (Fig. 5E), and eventually induced NPC apoptosis.

Chen et al. [36] revealed that the PERK/eIF2α pathway could promote autophagy; however, blocking the PERK/eIF2α pathway or inhibiting autophagy reduced NPC viability. Further experiments that supported the cellular protective effect of autophagy showed that H_2_O_2_ induced DNA damage in NPCs and caused AIM2-associated inflammatory cascades. After enhancing autophagy, autophagy-dependent secretion promotes AIM2 inflammasome secretion from NPCs, which inhibits cellular DNA damage and apoptosis, thus delaying the progression of IDD [18]. In our experiments, IGK significantly activated autophagy and protected NPCs by removing damaged organelles. A recent study confirmed that IGK can inhibit proteasome degradation to stabilize the Nrf2 protein, thus promoting nuclear translocation of Nrf2, which in turn triggers the activation of the antioxidant system and plays a role in protecting mitochondria [37]. In the dysfunctional mitochondria, more electrons leak from mitochondrial complexes, which subsequently generate H_2_O_2_ under the catalysis of superoxide dismutase [38]. Endogenous ROS can destroy intracellular biomacromolecules and cause cellular dysfunction or even death. As an autophagy stimulator, IGK can both resist mitochondrial collapse and induce impaired mitochondrial clearance; thus, it has powerful therapeutic potential in ROS-induced IDD.

Many studies have attempted to treat IDD using multidrug administration. This strategy allows the pharmacological effects of several drugs to be superimposed on each other and achieves greater therapeutic effects at lower drug concentrations [39–41]. A recent study revealed that dysfunction of either autophagy or antioxidant systems can lead to aggravated detrimental effects of H_2_O_2_ on intervertebral disc cells [42]. Therefore, we combined an autophagy stimulator (IGK) with ROS scavenger (SeNP) and found that the assembled nanodelivery system exerted superior efficacy. Moreover, an autophagy blocker attenuated the therapeutic effects of IGK@SeNP on ECM metabolism regulation and inhibition of apoptosis, indicating that IGK-induced autophagy is necessary for NPC self-repair. In addition, SeNP allows IGK to be more stably present within the disc, which is beneficial in reducing the frequency of medication and the complications of intradiscal injection. These characteristics make its clinical application promising.

However, we also considered the limitations of this study. Although we employed H_2_O_2_ to induce NPC degeneration, there may be more complex pathophysiological features in the actual degenerated discs [43]. Therefore, we will use NPCs obtained from patients with IDD for further validation. Moreover, the intervertebral disc is an avascular closed structure, and nanoparticles injected in situ are rarely distributed to other organs [31]. Thus, our study did not investigate blood compatibility and toxicity in vivo. Although HE staining of intervertebral discs showed no obvious inflammatory cell infiltration, we will verify the in vivo biocompatibility if IGK@SeNP is used in other disease models. Overall, we propose a novel ROS-responsive nanodrug delivery system that can delay the progression of IDD by synergistically enhancing autophagy and scavenging ROS.

## Conclusion

In this study, we synthesized a novel ROS-responsive nanodelivery system for SeNP loaded with IGK for IDD treatment. Our findings demonstrated that the nanoparticles had an ROS-stimulated responsive drug release effect. SeNP combined with IGK synergistically eliminated ROS and enhanced autophagy. IGK@SeNP protected NPCs and delayed the pathological process of IDD, both in vitro and in vivo. Collectively, this study suggests that IGK@SeNP may be an attractive therapeutic agent for treating IDD.

## Experimental section

### Materials and reagents

Selenium powder, sodium borohydride, isophorone diisocyanate (IPDI), methoxypolyethylene glycol (mPEG), dibutyltin dilaurate (DBTDL), 11-bromoundecanol, methoxypolyethylene glycols, Tetrahydrofuran (THF), hydrogen peroxide (H_2_O_2_), Cy 5 (Aladdin, Shanghai, China). Isoginkgetin (IGK), chloroquine (CQ), (MedChemExpress, Monmouth Junction, USA). Primary antibodies against aggrecan, collagen II, SOX9, MMP3, MMP13, ADAMTS5, ATG7, Beclin1, LC3B, SQSTM1/p62 (Abcam, Cambridge, UK), primary antibodies against BCL2, BAX and cleaved caspase-3 (Cell Signalling Technology, Danvers, USA), primary antibodies against β-actin (Proteintech, Wuhan, China). Goat anti-rabbit IgG H&L (Alexa Fluor^®^ 488), goat anti-rabbit IgG H&L (Alexa Fluor^®^ 594), (Abcam, Cambridge, UK), HRP-labeled goat anti-rabbit IgG (Proteintech, Wuhan, China). Penicillin–streptomycin (Sigma-Aldrich, St. Louis, USA). Cell counting kit-8 (CCK-8), Calcein/PI assay kit, ROS assay kit, and TUNEL apoptosis assay kit (Beyotime, Shanghai, China). Phosphate-buffered saline (PBS), FITC-annexin V/PI apoptosis detection kit, fetal bovine serum (FBS), DMEM/F12 (Thermo Fisher Scientific, Waltham, USA). Nitrocellulose membranes (Pall, New York, USA). Electrochemiluminescence substrate (Meilunbio, Dalian, China). Zoletile (Virvac, Carros, France).

### Synthesis of IGK@SeNP

The synthesis of SeNP was consistent with our previous work description [44]. In brief, selenium powder (1 g, 12.7 mmol) was dissolved in deionized water (15 mL) with stirring under N_2_ flow. Sodium borohydride solution (0.1 g/mL, 10 mL) was dropwise added to the reaction for 15 min. Secondly, another selenium powder (1 g, 12.7 mmol) was added and reacted for 15 min. Afterward, 11-bromoundecanol (6.33 g, 25.2 mmol) was dissolved in THF (25 mL) and added to the reaction system. The mixture was heated at 50 °C and maintained for 24 h, and then filtered while hot. The obtained solution was extracted three times with 20 mL of dichloromethane and dried with anhydrous sodium sulfate. The product was purified by column chromatography with a 1:4 mixture of ethyl acetate and methylene chloride as eluent. The di(1-hydroxylundecyl) diselenide was obtained after removing the solvent by rotary evaporation. Di(1-hydroxylundecyl) diselenide (2 g, 4 mmol) and IPDI (0.98 g, 4.40 mmol) were dissolved in THF (60 mL) under N_2_ flow. Then DBTDL (0.05 mg, 0.1 mmol) was added as a catalyst. After a reaction for 2 h at 50 °C, mPEG_2000_ (13 g, 6.50 mmol) was added and the reaction was continued at 50 °C for 24 h. THF was removed by rotary evaporation. The obtained Se-polymer was purified by cold methanol precipitated and collected after vacuum drying.

IGK (1 mg), triethylamine (50 μL), and Se-polymer (10 mg) were dissolved in N,N-dimethylformamide (DMF, 2 mL) and stirred for 2 h. Deionized water (10 mL) was slowly added with stirring. Subsequently, the solution was dialyzed for 24 h (MWCO 3500 Da) against deionized water. IGK-loaded nanoparticles (IGK@SeNP) were obtained by ultrafiltration centrifugation (MWCO 10000 Da). The SeNP and Cy5@SeNP were constructed using the same method. IGK@SeNP were freeze-dried and dissolved in DMSO, then detected using a UV−vis spectrometer. The IGK loading content was 8.69% and the drug entrapment efficiency was 41.78%.

### Characterization

^1^H NMR spectroscopy was used to detect the Se-polymer modified with IPDI and mPEG. The number-average molecular weight (M̅n) and weight-average molecular weights (M̅w) of the Se-polymers were determined using gel permeation chromatography (GPC). The morphology of SeNP was observed and reported using a transmission electron microscope (TEM) at an acceleration voltage of 120 kV. The particle diameters and dispersion of particle size were reported by dynamic light scattering (DLS).

### ROS-responsive drug release

5 mL of Cy5@SeNP solution was treated with gradient concentrations of H_2_O_2_ (0, 50, 100, 200 μM). The Cy5 content was measured using a fluorescence spectrophotometer at 650 nm excitation wavelength.

### Cell culture and treatment

Human nucleus pulposus cells (NPCs, ScienCell, Carlsbad, USA) were cultured in DMEM/F12 medium supplemented with 10% FBS, 1% penicillin–streptomycin at 37 °C and 5% CO2. To simulate an oxidative microenvironment of degenerative disc, NPCs were pretreated with PBS, IGK, SeNP, or IGK@SeNP for 4 h, then stimulated with 200 μM H_2_O_2_ for 24 h.

### Cellular uptake

Due to the lack of fluorescent properties of IGK, we used Cy5 as a drug model to investigate the cellular uptake of Cy5@SeNP in NPCs [45]. NPCs were seeded in 24-well plates. After washes with PBS, Hoechst 33342 was incubated with the cells for 30 min at 37 °C. Subsequently, the culture medium contained Cy5@SeNP replaced the original medium. The cellular uptake was observed by fluorescence microscope.

### Live/dead staining assay

NPCs were seeded into 24-well plates and co-cultured with IGK@SeNP (60 μg/mL). After washing with PBS, the Calcein/PI staining dye was added and incubated for 30 min at 37 °C for 24, 48, and 72 h. After incubation, the cells were washed again and observed under a fluorescence microscope.

### ROS-scavenging evaluation

Intracellular ROS level was detected by DCFH-DA. NPCs were cultured in 6-well plates and were treated as per the aforementioned protocol. Afterward, the cells were stained with DCFH-DA (10 μM) for 20 min at 37 °C and washed 3 times with serum-free DMEM/F12. The flow cytometer analysis was executed by FACSVerse.

### Rat IDD model and treatment

All animal experiments were performed following the Ethics Committee for Animal Experiments. A total of sixty 8-week-old male SD rats were randomly allocated to 5 groups: Control, IDD, IGK, SeNP, and IGK@SeNP. The IDD model was established by needle puncture [46]. Rats were intraperitoneally anesthetized with 50 mg/kg Zoletile and 3 mg/kg xylazine hydrochloride. After successful anesthesia, the rats were placed prone on a clean operating table. The rat tails were disinfected and draped, and a 20G needle was used to penetrate 5 mm percutaneously into the dorsal center on Co 7/8 and Co 8/9. The needle was rotated 360° and removed after staying for 30 s. The surgical area was disinfected again to avoid infection.

To determine whether the modeling was successful, a magnetic resonance imaging (MRI) examination was conducted after 2 weeks. The degenerative discs were orthotopically injected with PBS (2 μL), IGK (2 μL, 20 μM), SeNP (2 μL, 60 μg/mL), or IGK@SeNP (2 μL, 60 μg/mL) using a 33G needle.

### Radiology evaluation

X-rays and MRI were performed at 4 and 8 weeks after IDD construction. The anesthetized rats were placed supine and the tails were straightened. The intervertebral heights were measured by image J on X-ray images. A 1.5 T MRI scanner was used to acquire the intervertebral disc signals on T2 weighted phase. Further, the severity of IDD was assessed according to the modified Pfirrmann grading system [47]

### Histological analysis

The rats were sacrificed by injecting over a dose of pentobarbital sodium at 4 weeks or 8 weeks. Intervertebral discs were harvested under aseptic conditions and fixed in 4% paraformaldehyde for 24 h. After decalcification by 10% EDTA for 2 weeks, the discs were sliced into 4 μm thick sections. Hematoxylin–eosin (HE), safranin O-fast green (SF), immunochemical (IHC), and IF staining were performed according to the manufacturer’s instructions [48, 49]. The morphology of intervertebral discs was observed under a light microscope and determined the histological grade [50].

### Statistical analysis

All statistical analyses were conducted with SPS software version 26.0 (IBM SPSS Corp. Chicago, USA). Data were presented as mean ± standard deviations (SD). Student’s t-test or one-way analysis of variance (ANOVA) was used to evaluate the differences among various treatment groups. P < 0.05 was considered statistically significant. (*, †, and # denoted P < 0.05).

## Supplementary Information

**Additional file 1: Fig S1.** ^1^H NMR characterization of di(1-hydroxylundecyl) diselenide in CDCl_3_. **Fig S2.** GPC plot of diselenide-containing polymer in DMF.

## Data Availability

The datasets generated and/or analyzed during the current study are not publicly available but are available from the corresponding author on reasonable request.

## Abbreviations

IDD: Intervertebral disc degeneration
ECM: Extracellular matrix
NPCs: Nucleus pulposus cells
IGK: Isoginkgetin
CQ: Chloroquine
Cy5: Cyanine5
ROS: Reactive oxygen species
H_2_O_2_: Hydrogen peroxide
SeNP: Diselenide-containing nanoparticles
IGK@SeNP: Isoginkgetin-loaded diselenide-containing nanoparticles
Se-polymer: Diselenide-containing polymer
IPDI: Diisocyanate
DBTDL: Dibutyltin dilaurate
mPEG: Methoxypolyethylene glycol (mPEG)
THF: Tetrahydrofuran
PBS: Phosphate-buffered saline
FBS: Fetal bovine serum
EDTA: Ethylene diamine tetraacetic acid
TEM: Transmission electron microscopy
DLS: Dynamic light scattering
gMFI: Geometric mean fluorescence intensity
COL2: Collagen II
SOX9: Sex-determining region Y-type high mobility group box protein 9;
ACAN: Aggrecan
MMP3: Matrix metalloproteinases 3
MMP13: Matrix metalloproteinases 13
ADAMTS5: A disintegrin and metalloproteinase with thrombospondin motifs 5
BCL2: B-cell lymphoma 2
BAX: BCL2 associated X
CASP3: Caspase 3
ATG7: Autophagy related 7
BECN1: Beclin 1
LC3: Microtubule associated protein 1 light chain 3
SQSTM1/p62: Sequestosome 1
CCK8: Cell counting kit-8
TUNEL: Terminal-deoxynucleoitidyl transferase mediated nick end labeling
MRI: Magnetic resonance imaging
HE: Hematoxylin–eosin
SF: O-fast green
IF: Immunofluorescence
IHC: Immunochemistry

## References

[1] Knezevic NN, Candido KD, Vlaeyen JWS, Van Zundert J, Cohen SP (2021). Low back pain. Lancet.

[2] Francisco V, Pino J, Gonzalez-Gay MA, Lago F, Karppinen J, Tervonen O, Mobasheri A, Gualillo O (2022). A new immunometabolic perspective of intervertebral disc degeneration. Nat Rev Rheumatol.

[3] Alvarez-Garcia O, Matsuzaki T, Olmer M, Miyata K, Mokuda S, Sakai D, Masuda K, Asahara H, Lotz MK (2018). FOXO are required for intervertebral disk homeostasis during aging and their deficiency promotes disk degeneration. Aging Cell.

[4] Che H, Li J, Li Y, Ma C, Liu H, Qin J, Dong J, Zhang Z, Xian CJ, Miao D (2020). p16 Deficiency attenuates intervertebral disc degeneration by adjusting oxidative stress and nucleus pulposus cell cycle. Elife.

[5] Zhang W, Li G, Luo R, Lei J, Song Y, Wang B, Ma L, Liao Z, Ke W, Liu H (2022). Cytosolic escape of mitochondrial DNA triggers cGAS-STING-NLRP3 axis-dependent nucleus pulposus cell pyroptosis. Exp Mol Med.

[6] Yu BP (1994). Cellular defenses against damage from reactive oxygen species. Physiol Rev.

[7] Kang L, Liu S, Li J, Tian Y, Xue Y, Liu X (2020). The mitochondria-targeted anti-oxidant MitoQ protects against intervertebral disc degeneration by ameliorating mitochondrial dysfunction and redox imbalance. Cell Prolif.

[8] Grunhagen T, Shirazi-Adl A, Fairbank JC, Urban JP (2011). Intervertebral disk nutrition: a review of factors influencing concentrations of nutrients and metabolites. Orthop Clin North Am.

[9] Colella F, Garcia JP, Sorbona M, Lolli A, Antunes B, D'Atri D, Barre FPY, Oieni J, Vainieri ML, Zerrillo L (2020). Drug delivery in intervertebral disc degeneration and osteoarthritis: selecting the optimal platform for the delivery of disease-modifying agents. J Control Release.

[10] Haro H, Ebata S, Inoue G, Kaito T, Komori H, Ohba T, Sakai D, Sakai T, Seki S, Shiga Y (2022). Japanese Orthopaedic Association (JOA) clinical practice guidelines on the management of lumbar disc herniation, third edition—secondary publication. J Orthop Sci.

[11] Pope MH (1989). Biomechanics of the lumbar spine. Ann Med.

[12] Liu T, Xiao B, Xiang F, Tan J, Chen Z, Zhang X, Wu C, Mao Z, Luo G, Chen X, Deng J (2020). Ultrasmall copper-based nanoparticles for reactive oxygen species scavenging and alleviation of inflammation related diseases. Nat Commun.

[13] Li X, Liu Y, Qi X, Xiao S, Xu Z, Yuan Z, Liu Q, Li H, Ma S, Liu T (2022). Sensitive activatable nanoprobes for real-time ratiometric magnetic resonance imaging of reactive oxygen species and ameliorating inflammation in vivo. Adv Mater.

[14] Yang L, Yu C, Fan X, Zeng T, Yang W, Xia J, Wang J, Yao L, Hu C, Jin Y (2022). Dual-dynamic-bond cross-linked injectable hydrogel of multifunction for intervertebral disc degeneration therapy. J Nanobiotechnol.

[15] Zhang Z, Xu T, Chen J, Shao Z, Wang K, Yan Y, Wu C, Lin J, Wang H, Gao W (2018). Parkin-mediated mitophagy as a potential therapeutic target for intervertebral disc degeneration. Cell Death Dis.

[16] Hu S, Zhang C, Qian T, Bai Y, Chen L, Chen J, Huang C, Xie C, Wang X, Jin H (2021). Promoting Nrf2/Sirt3-dependent mitophagy suppresses apoptosis in nucleus pulposus cells and protects against intervertebral disc degeneration. Oxid Med Cell Longev.

[17] Levine B, Mizushima N, Virgin HW (2011). Autophagy in immunity and inflammation. Nature.

[18] Li S, Liao Z, Luo R, Song Y, Wang K, Feng X, Ou Y, Wu X, Zhang Y, Gao Y (2021). Autophagy-based unconventional secretory for AIM2 inflammasome drives DNA damage resistance during intervertebral disc degeneration. Front Cell Dev Biol.

[19] He R, Wang Z, Cui M, Liu S, Wu W, Chen M, Wu Y, Qu Y, Lin H, Chen S (2021). HIF1A alleviates compression-induced apoptosis of nucleus pulposus derived stem cells via upregulating autophagy. Autophagy.

[20] Shao Z, Ni L, Hu S, Xu T, Meftah Z, Yu Z, Tian N, Wu Y, Sun L, Wu A (2021). RNA-binding protein HuR suppresses senescence through Atg7 mediated autophagy activation in diabetic intervertebral disc degeneration. Cell Prolif.

[21] Liu L, Wang Y, Zhang J, Wang S (2021). Advances in the chemical constituents and chemical analysis of ginkgo biloba leaf, extract, and phytopharmaceuticals. J Pharm Biomed Anal.

[22] Yao J, Tang S, Shi C, Lin Y, Ge L, Chen Q, Ou B, Liu D, Miao Y, Xie Q (2022). Isoginkgetin, a potential CDK6 inhibitor, suppresses SLC2A1/GLUT1 enhancer activity to induce AMPK-ULK1-mediated cytotoxic autophagy in hepatocellular carcinoma. Autophagy.

[23] He Y, Li W, Zheng Z, Zhao L, Li W, Wang Y, Li H (2020). Inhibition of protein arginine methyltransferase 6 reduces reactive oxygen species production and attenuates aminoglycoside- and cisplatin-induced hair cell death. Theranostics.

[24] Dong J, Zhang KJ, Li GC, Chen XR, Lin JJ, Li JW, Lv ZY, Deng ZZ, Dai J, Cao W, Jiang Q (2022). CDDO-im ameliorates osteoarthritis and inhibits chondrocyte apoptosis in mice via enhancing Nrf2-dependent autophagy. Acta Pharmacol Sin.

[25] Yuan L, Zhang F, Qi X, Yang Y, Yan C, Jiang J, Deng J (2018). Chiral polymer modified nanoparticles selectively induce autophagy of cancer cells for tumor ablation. J Nanobiotechnol.

[26] Peng Z, Yuan L, XuHong J, Tian H, Zhang Y, Deng J, Qi X (2021). Chiral nanomaterials for tumor therapy: autophagy, apoptosis, and photothermal ablation. J Nanobiotechnol.

[27] Mizushima N, Yoshimori T, Levine B (2010). Methods in mammalian autophagy research. Cell.

[28] Kim HS, Wu PH, Jang IT (2020). Lumbar degenerative disease part 1: anatomy and pathophysiology of intervertebral discogenic pain and radiofrequency ablation of basivertebral and sinuvertebral nerve treatment for chronic discogenic back pain: a prospective case series and review of literature. Int J Mol Sci.

[29] Zorov DB, Juhaszova M, Sollott SJ (2014). Mitochondrial reactive oxygen species (ROS) and ROS-induced ROS release. Physiol Rev.

[30] Yin H, Wang K, Das A, Li G, Song Y, Luo R, Cheung JPY, Zhang T, Li S, Yang C (2021). The REDD1/TXNIP complex accelerates oxidative stress-induced apoptosis of nucleus pulposus cells through the mitochondrial pathway. Oxid Med Cell Longev.

[31] Hickman TT, Rathan-Kumar S, Peck SH (2022). Development, pathogenesis, and regeneration of the intervertebral disc: current and future insights spanning traditional to omics methods. Front Cell Dev Biol.

[32] Lim S, An SB, Jung M, Joshi HP, Kumar H, Kim C, Song SY, Lee JR, Kang M, Han I, Kim BS (2022). Local delivery of senolytic drug inhibits intervertebral disc degeneration and restores intervertebral disc structure. Adv Healthc Mater.

[33] Cheng YH, Yang SH, Lin FH (2011). Thermosensitive chitosan-gelatin-glycerol phosphate hydrogel as a controlled release system of ferulic acid for nucleus pulposus regeneration. Biomaterials.

[34] Zhu L, Yang Y, Yan Z, Zeng J, Weng F, Shi Y, Shen P, Liu L, Yang H (2022). Controlled release of TGF-β3 for effective local endogenous repair in IDD using rat model. Int J Nanomed.

[35] Bai J, Zhang Y, Fan Q, Xu J, Shan H, Gao X, Ma Q, Sheng L, Zheng X, Cheng W (2020). Reactive oxygen species-scavenging scaffold with rapamycin for treatment of intervertebral disk degeneration. Adv Healthc Mater.

[36] Chen L, Liu L, Xie ZY, Wang F, Zhu L, Zhang C, Fan P, Sinkemani A, Hong X, Wu XT (2019). Protein kinase RNA-like ER kinase/eukaryotic translation initiation factor 2α pathway attenuates tumor necrosis factor alpha-induced apoptosis in nucleus pulposus cells by activating autophagy. J Cell Physiol.

[37] Wu X, Huang J, Tang J, Sun Y, Zhao G, Yan C, Liu Z, Yi W, Xu S, Yu X (2022). Isoginkgetin, a bioactive constituent from ginkgo biloba, protects against obesity-induced cardiomyopathy via enhancing Nrf2/ARE signaling. Redox Biol.

[38] Filomeni G, De Zio D, Cecconi F (2015). Oxidative stress and autophagy: the clash between damage and metabolic needs. Cell Death Differ.

[39] Cho H, Lee S, Park SH, Huang J, Hasty KA, Kim SJ (2013). Synergistic effect of combined growth factors in porcine intervertebral disc degeneration. Connect Tissue Res.

[40] Colombier P, Clouet J, Boyer C, Ruel M, Bonin G, Lesoeur J, Moreau A, Fellah BH, Weiss P, Lescaudron L (2016). TGF-β1 and GDF5 act synergistically to drive the differentiation of human adipose stromal cells toward nucleus pulposus-like cells. Stem Cells.

[41] Yu C, Li D, Wang C, Xia K, Wang J, Zhou X, Ying L, Shu J, Huang X, Xu H (2021). Injectable kartogenin and apocynin loaded micelle enhances the alleviation of intervertebral disc degeneration by adipose-derived stem cell. Bioact Mater.

[42] Kang L, Liu S, Li J, Tian Y, Xue Y, Liu X (2020). Parkin and Nrf2 prevent oxidative stress-induced apoptosis in intervertebral endplate chondrocytes via inducing mitophagy and anti-oxidant defenses. Life Sci.

[43] Huang YC, Leung VY, Lu WW, Luk KD (2013). The effects of microenvironment in mesenchymal stem cell-based regeneration of intervertebral disc. Spine J.

[44] Zhang L, Zhang S, Xu J, Li Y, He J, Yang Y, Huynh T, Ni P, Duan G, Yang Z, Zhou R (2020). Low-dose X-ray-responsive diselenide nanocarriers for effective delivery of anticancer agents. ACS Appl Mater Interfaces.

[45] Yang G, Fan M, Zhu J, Ling C, Wu L, Zhang X, Zhang M, Li J, Yao Q, Gu Z, Cai X (2020). A multifunctional anti-inflammatory drug that can specifically target activated macrophages, massively deplete intracellular H(2)O(2), and produce large amounts CO for a highly efficient treatment of osteoarthritis. Biomaterials.

[46] Ge J, Yan Q, Wang Y, Cheng X, Song D, Wu C, Yu H, Yang H, Zou J (2020). IL-10 delays the degeneration of intervertebral discs by suppressing the p38 MAPK signaling pathway. Free Radic Biol Med.

[47] Qian J, Ge J, Yan Q, Wu C, Yang H, Zou J (2019). Selection of the optimal puncture needle for induction of a rat intervertebral disc degeneration model. Pain Physician.

[48] Ge J, Cheng X, Yan Q, Wu C, Wang Y, Yu H, Yang H, Zhou F, Zou J (2020). Calcitonin inhibits intervertebral disc degeneration by regulating protein kinase C. J Cell Mol Med.

[49] Xu X, Wang D, Zheng C, Gao B, Fan J, Cheng P, Liu B, Yang L, Luo Z (2019). Progerin accumulation in nucleus pulposus cells impairs mitochondrial function and induces intervertebral disc degeneration and therapeutic effects of sulforaphane. Theranostics.

[50] Han B, Zhu K, Li FC, Xiao YX, Feng J, Shi ZL, Lin M, Wang J, Chen QX (2008). A simple disc degeneration model induced by percutaneous needle puncture in the rat tail. Spine.

