# Supplementary material for: Isoginkgetin-loaded reactive oxygen species scavenging nanoparticles ameliorate intervertebral disc degeneration via enhancing autophagy in nucleus pulposus cells
Source: J Nanobiotechnology. 2023 Mar 21;21:99. doi: 10.1186/s12951-023-01856-9 (PMC10029295; doi:10.1186/s12951-023-01856-9)
Supplement: Supplementary file 1 — Additional file 1: Fig S1. 1H NMR characterization of di(1-hydroxylundecyl) diselenide in CDCl3. Fig S2. GPC plot of diselenide-containing polymer in DMF. [file 12951_2023_1856_MOESM1_ESM.docx]

# Experimental section

## Instruments

^1^H NMR spectroscopy (INOVA-400, Varian, Palo Alto, USA), Gel permeation chromatograph (HLC-8320, TOSOH, Tokyo, Japan), Transmission electron microscope (HT7700, Hitachi, Tokyo, Japan), Dynamic light scattering granulometer (ZetaSizer Nano ZS90, Malvern Panalytical, Malvern, UK), Fluorescence spectrophotometer (Cary Eclipse, Agilent Technologies, Palo Alto, USA), Fluorescence microscope (DM i8, Leica, Weztlar, Germany), Laser-scanning confocal microscope (FV1200, Olympus, Tokyo, Japan), Microplate reader (Synergy 2, BioTek, Winooski, USA), Flow cytometry (FACSVerse, BD Biosciences, Franklin Lakes, USA), Transmission Electron Microscopy (Tecnai G2 20 TWIN, FEI, Hillsboro, USA)

## Cytotoxicity assay

CCK-8 assay was utilized to assess the cytotoxicity of SeNPs and IGK@SeNPs against NPCs. Briefly, NPCs were seeded into 96-well plates within 100 μL cell suspension containing 5000 cells per well. Then various concentrations (0, 20, 40, 60, 80, 100 μg/mL) nanoparticles were added into the culture medium and co-cultured with NPCs for 24, 48, and 72 h, respectively. The culture medium was replaced with serum-free DMEM/F12 containing 10% CCK-8 and incubated for 1 hour. The OD values at 450 nm were analyzed by a microplate reader.

## Western blotting

After NPCs were treated as described above, the proteins were extracted by RIPA Lysis Buffer. Samples with the equivalent of protein were separated with 10-15% SDS-PAGE gels and transferred to nitrocellulose membranes. The membranes were blocked and incubated with primary antibodies (1:1000–1:3000) at 4 °C overnight, then incubated with HRP-labeled secondary antibodies (1:10000) at room temperature for 1 h. Protein bands were visualized using an electrochemiluminescence substrate.

**Apoptosis rate analysis**

NPCs were seeded in 6-well plates and treated as described above. The cells from each well were collected into separate EP tubes and resuspended using 100 μL binding buffer. Afterward, 5 μL Annexin V-FITC and 5 μL PI were added into every tube and incubated at room temperature for 15 minutes. Eventually, 400 μL binding buffer was added into every tube and mixed well. The samples were detected using flow cytometry.

**TUNEL staining**

The treated NPCs in 24-well plates were fixed using 4% paraformaldehyde and permeated by PBS containing 0.3% Triton. 50μl TUNEL detection solution was added and incubated for 1 h at 37ºC. The cell nucleus was stained with DAPI. The fluorescence images were captured under a fluorescence microscope.

**Immunofluorescence**

NPCs were first accepted various treatments, then were fixed for 15 min and permeated for 5 min. After 1 hour of blocking, the cells were incubated with primary antibodies (1:200) at 4°C overnight, then incubated with fluorescence secondary antibodies (1:600) at room temperature for 1 h. The cell nucleus was stained with DAPI. Images were obtained with a fluorescence microscope and a laser-scanning confocal microscope.

**Transmission Electron Microscopy**

The cell masses were then fixed in 2.5% glutaraldehyde, then were post-fixed with 1% osmium tetroxide for 1 h. Subsequently, dewatering with acetone. After being embedded with Eponate 12, the samples were sliced into 0.5 μm thick slices and stained with toluidine blue. Finally, ultrathin sections of 50 nm were cut and stained with uranyl acetate and lead citrate in the dark. The images were obtained by TEM.


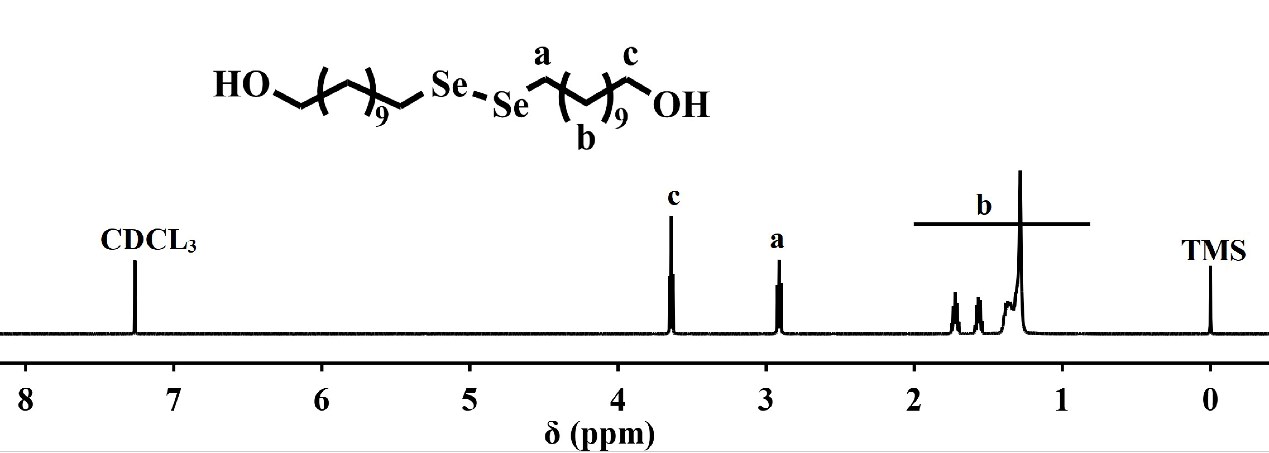


# Fig. S1 ^1^H NMR characterization of di(1-hydroxylundecyl) diselenide in CDCl_3_.


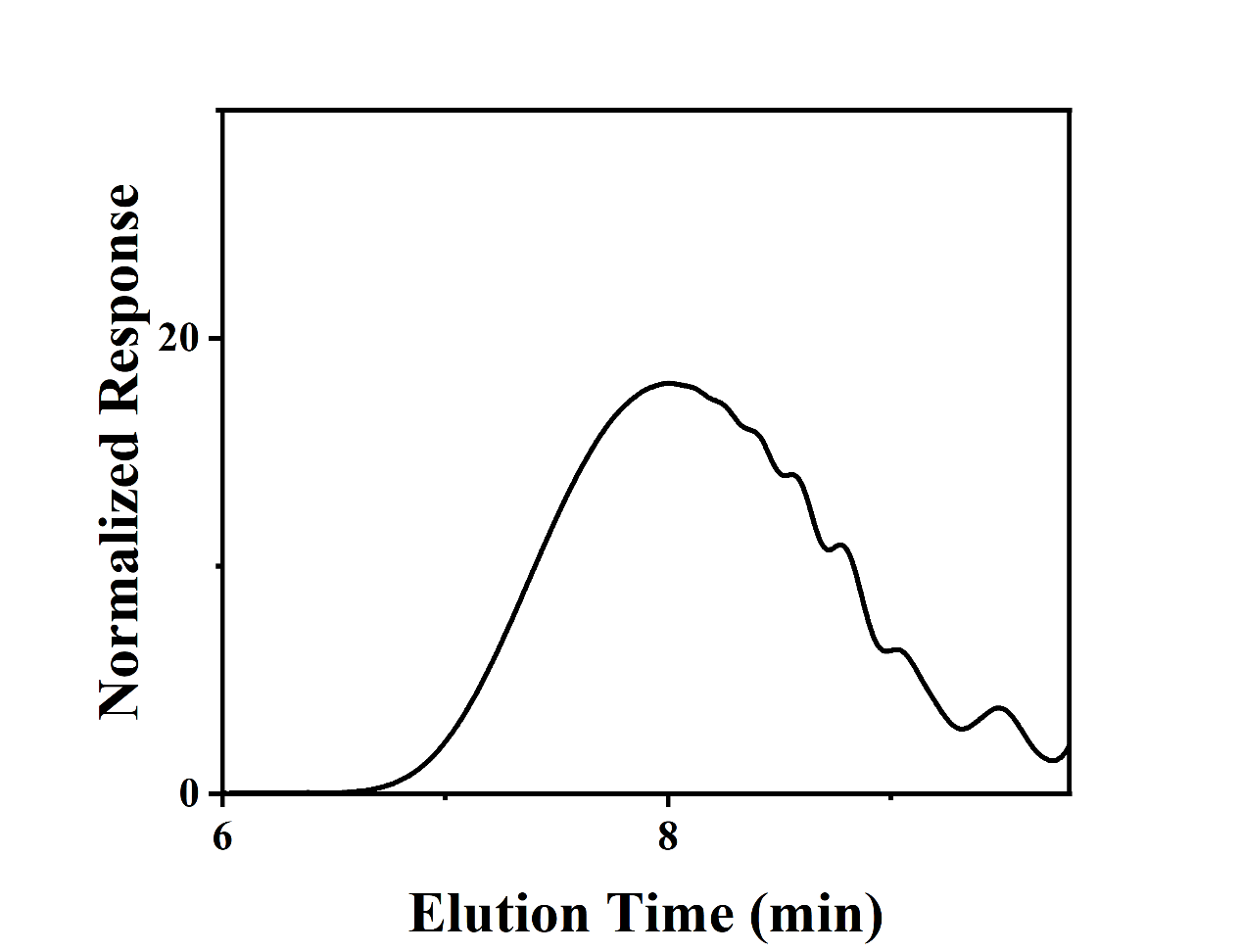


# Fig. S2 GPC plot of diselenide-containing polymer in DMF.
